# Supplementary material for: Redundant representations are required to disambiguate simultaneously presented complex stimuli
Source: PLoS Comput Biol. 2023 Aug 9;19(8):e1011327. doi: 10.1371/journal.pcbi.1011327 (PMC10442167; doi:10.1371/journal.pcbi.1011327)
Supplement: S1 Text — In the supplementary text, we include additional detail on several points discussed in the main text as well as explore several related topics. In particular, we Consider the case of asymmetric feature representations.Consider the case of nonlinear mappings between the two feature representations.Derive an expression for the variance of spiking energy of the RF codes. (PDF) [file pcbi.1011327.s001.pdf]

S1 S1 Text

S1 Asymmetric feature encoding

In general, the representations of the same object feature may not be encoded with the same fidelity in each region. For instance, estimates of azimuthal position from the visual system are thought to be much more accurate than those from the auditory system[1]. Further, the relative accuracy of estimates of the azimuthal position and elevation of a particular sound shifts across the sensory field[2]. Here, we show how these asymmetric feature representations affect the assignment error rate. In all cases, we assume that the two sources of stimulus information are optimally combined by the downstream brain region, such that the estimator variance of a commonly represented feature D_S is given by the following relationship,

$$D_S = \frac{D_X D_Y}{D_X + D_Y}$$

where D_X and D_Y are, as before, the estimator variance of the representation of that feature in regions R_X and R_Y, respectively (Fig AA). Using this equation for the integrated estimator variance, we can rewrite D_X and D_Y in terms of the integrated estimator variance D_S and the representation asymmetry ΔD,

$$D_X = \frac{2D_S}{1 - \Delta D}$$
$$D_Y = \frac{2D_S}{1 + \Delta D}$$

where, without loss of generality, we have assumed $D_Y \geq D_X$ (Fig AB) and the representation asymmetry ΔD is constrained to be on the interval $[0, 1)$. When $\Delta D = 0$, the feature representations are perfectly symmetric, as before, and $D_X = D_Y = 2D_S$; when $\Delta D \to 1$, then $D_X \to D_S$ and $D_Y \to \infty$. A fully asymmetric representation provides no redundancy and thus cannot be used to solve the assignment problem, effectively reducing the number of commonly represented features C.

Further, we show that increasing representation asymmetry increases the assignment error rate, both for all individual distances between stimuli δ (Fig AC) and overall (Fig AD). That is, increasing the level of asymmetry while keeping other features of the representations constant increases the assignment error rate. Next, we evaluate the redundancy between $\hat{X}$ and $\hat{Y}$ as a function of precision ratio and representation asymmetry. As expected, increasing representation asymmetry also decreases the level of redundancy between the two sets of representations (Fig AE), as it makes the set of representations observed in one region less informative about the representations observed in the other.

In some cases, asymmetric feature representations are imposed by the physical limits of different sensory systems, such as with audition and vision; in others, however, asymmetric feature representations can be designed, such as is hypothesized to be the case across the canonical dorsal and ventral visual processing streams in the primate. In that case, representations of position are thought to be encoded with high fidelity in the dorsal visual stream, but with relatively low fidelity in the ventral visual stream. Yet, the assignment problem must also be solved when integrating across those two sets of regions, so an asymmetric representation of spatial position across them may seem non-optimal. Next, we resolve this apparent contradiction by showing that, in some cases, asymmetric feature representations increase the efficiency of solutions to the assignment problem by reducing the redundancy between the two regions.

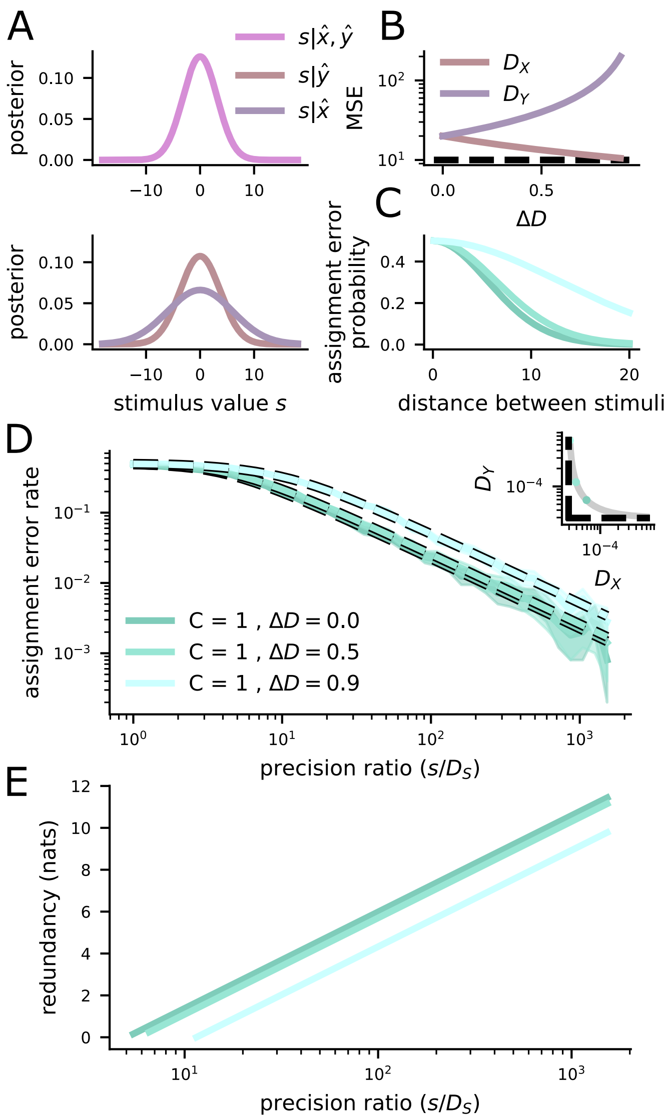

Fig A: Asymmetric feature representations increase the assignment error rate, but decrease redundancy. **A** Schematic of representation integration. The stimulus estimates from R_X and R_Y are optimally combined to give rise to a stimulus estimate with variance $D_S = D_X D_Y/(D_X + D_Y)$. **B** We can keep D_S constant (dashed line) and define D_X and D_Y in terms of D_S and the representation asymmetry ΔD, which is 0 when $D_X = D_Y$ and goes to 1 when $D_X \to D_S$ and $D_Y \to \infty$, or vice versa. **C** Increasing representation asymmetry ΔD increases the assignment error rate for pairs of stimuli at all distances. **D** The overall assignment error rate increases with increases in representation asymmetry across all precision ratios. **E** The redundancy between $\hat{X}$ and $\hat{Y}$ decreases with increases in representation asymmetry across all precision ratios.

S1.1 Representation asymmetry increases the assignment error rate

For asymmetric local distortion, **??** can be rewritten as

$$F(\delta, \Delta D) \approx Q\left(\frac{-\delta\sqrt{1-\Delta D}}{\sqrt{4D_S}}\right) + Q\left(\frac{-\delta\sqrt{1+\Delta D}}{\sqrt{4D_S}}\right)$$

which reveals that one term of the sum will decrease with increased asymmetry and the other term will increase. Yet, it is not clear how this should affect the assignment error rate at a particular δ. To resolve this, we study the derivative of $F(\delta, \Delta D)$ with respect to ΔD,

$$\frac{\partial}{\partial \Delta D} F(\delta, \Delta D) \approx \frac{\delta}{4\sqrt{D_S}}\left(f\left(\frac{-\delta\sqrt{1-\Delta D}}{\sqrt{4D_S}}\right) - f\left(\frac{-\delta\sqrt{1+\Delta D}}{\sqrt{4D_S}}\right)\right)$$

where f is the probability density function of the standard Gaussian. From this, we can see that the first term will always be larger than or the same size (when $\Delta D = 0$) as the second term. Thus, the assignment error rate will increase as ΔD increases.

S2 Nonlinear mappings between $\hat{X}$ and $\hat{Y}$

In the main text, we consider representations in R_X and R_Y that directly share one or more features (i.e., the features are identical, Fig B, left). Alternatively, some of the features encoded by R_Y could have a linear relationship with some of the features encoded by R_X, such that:

$$\hat{Z}_C = MY + b + \epsilon$$

where Y is the true values of all the features encoded in R_Y, $\hat{Z}_C$ is the estimate of the common features encoded by R_X, M is a linear transform, b is an intercept, and $\epsilon \sim \mathcal{N}(0, D_Y)$. So, we can then perform assignment by comparing the common features decoded from R_X, $\hat{X}_C$ and the common features decoded from Y into R_X's frame of reference, $\hat{Z}_C$. In this case, we are comparing quantities with D_X and D_Y as in the main text, though these two quantities need not be the same.

However, in principle, the dependence between $\hat{X}$ and $\hat{Y}$ could be nonlinear (Fig B, right). There are two main cases here. First,

$$\hat{Z}_C = f(Y) + \epsilon$$

where the function $f(.)$ is a nonlinear decoder for the common features from R_Y. In this case, the noise is assumed to be uniform across different values of Y. Here, the equations that we develop apply directly, since we can account for different noise levels between $\hat{X}_C$ and $\hat{Z}_C$ by differing D_X and D_Y.

In contrast, the second case would be that in which,

$$\hat{Z}_C = f(Y) + \epsilon(Y)$$

where the noise also depends on the value of the common feature. We can still approach this case using our framework, but the integrals that we are required to evaluate are unlikely to be tractable or easily approximable except for very simple noise dependencies.

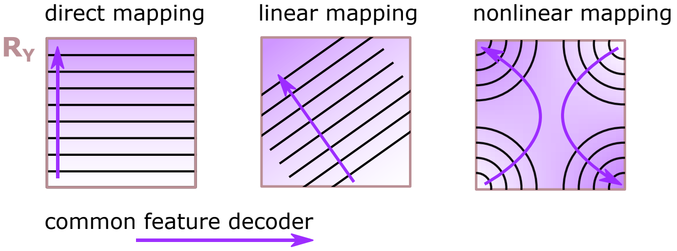

Fig B: Different mappings between the features in R_X and R_Y. (left) A direct mapping where the two regions encode the same features. (center) A linear mapping, where the two regions encode slightly different features, but where the features can be related by a linear map. (right) A nonlinear mapping, where the two regions encode different features, but that have a nonlinear dependence with each other.

S3 Variance of the spiking energy V of the random RF codes

Due to the random placement of the receptive fields, there will tend to be significant variance in V across different stimulus values. This variance is important for quantifying threshold errors. So,

$$\mathrm{Var}_x(V) = \mathbb{E}_x\left[V^2\right] - \mathbb{E}_x[V]^2$$

where the second term is already given above. For the first term,

$$\begin{aligned}\mathbb{E}_x\left[V^2\right] &= \mathbb{E}_x\left[\left(\sum_i^N r_i^2(x)\right)^2\right] \\ &= \sum_i^N \int_0^1 dx_1 ... \int_0^1 dx_D p(x) r_i^4(x) + \sum_{i\neq j}^{N(N-1)} \int_0^1 dx_1 ... \int_0^1 dx_D p(x) r_i^2(x) r_j^2(x)\end{aligned}$$

Here, the first sum follows along the same lines as above, where for a particular i,

$$\begin{aligned}\int_0^1 dx_1 ... \int_0^1 dx_D p(x) r_i^4(x) &= P^4 \prod_j^D \int_0^1 dx_j p(y_j) \exp\left[-\frac{2y_j^2}{w^2}\right] \\ &= 2P^4 \prod_j^D \int_0^1 dx_j (1-y_j) \exp\left[-\frac{2y_j^2}{w^2}\right] \\ &= P^4 \prod_j^D \sqrt{\frac{\pi}{2}} w\mathrm{erf}\left(\frac{\sqrt{2}}{w}\right) - \frac{w^2}{2}\left(1 - \exp\left[-\frac{2}{w^2}\right]\right)\end{aligned}$$

where the integral is given as above when changing w for $w/\sqrt{2}$.

The second term requires some additional trickery. We proceed for particular units i and h along dimension j, where we notice that $p(y_{ij})$ and $p(y_{hj})$ are not independent. Intuitively, if y_{ij} is large, then this must mean that x_j is near the edge of the stimulus space (i.e., near 0 or 1) and that implies that y_{hj} can take on large values too. To evaluate the integral, we need to decouple these two probabilities. So, we (re-)introduce x_j such that $p(y_{hj}|x_j)p(x_j) = p(y_{hj})$. So, we need to evaluate the triple integral (for particular units h and i, along dimension j),

$$P^4 \int_0^1 dx_j \int_{-x_j}^{1-x_j} dy_i \exp\left[-\frac{2y_i^2}{w^2}\right] \int_{-x_j}^{1-x_j} dy_h \exp\left[-\frac{2y_h^2}{w^2}\right]$$

where now the integrals are over a uniform distribution. So, we obtain,

$$P^4 \int_0^1 dx_j p(x_j) \left[\frac{1}{2}\sqrt{\pi} w \left(\mathrm{erf}\left(\frac{1-x_j}{w} \right) + \mathrm{erf}\left(\frac{x_j}{w} \right) \right) \right]^2$$

We can expand this into three separate terms. All of the terms can be integrated except one, and that one can be approximated for moderately small w.

First,

$$\begin{aligned} \int_0^1 dx_j \mathrm{erf}^2\left(\frac{1-x_j}{w} \right) &= \int_0^1 dx_j \mathrm{erf}^2\left(\frac{x_j}{w} \right) \\ &= \frac{2}{\sqrt{\pi}} w \exp\left[-1/w^2 \right] \mathrm{erf}\left(1/w \right) + \mathrm{erf}^2\left(1/w \right) - \sqrt{\frac{2}{\pi}} w \mathrm{erf}\left(\sqrt{2}/w \right) \end{aligned}$$

and, second,

$$\begin{aligned} \int_0^1 dx_j \mathrm{erf}\left(\frac{1-x_j}{w} \right) \mathrm{erf}\left(\frac{x_j}{w} \right) &\approx \int_0^{.5} dx_j \mathrm{erf}\left(\frac{x_j}{w} \right) + \int_{.5}^1 dx_j \mathrm{erf}\left(\frac{1-x_j}{w} \right) \\ &= 2\int_0^{.5} dx_j \mathrm{erf}\left(\frac{x_j}{w} \right) \\ &= \mathrm{erf}\left(\frac{1}{2w} \right) - \frac{2w}{\sqrt{\pi}} \left[1 - \exp\left[-\frac{1}{4w^2} \right] \right] \end{aligned}$$

So,

$$\begin{aligned} \mathbb{E}_x\left[V^2 \right] =& N P^4 \prod_j^D \left[\sqrt{\frac{\pi}{2}} w \mathrm{erf}\left(\frac{\sqrt{2}}{w} \right) - \frac{w^2}{2} \left(1 - \exp\left[-\frac{2}{w^2} \right] \right) \right] \\ &+ N(N-1) P^4 \prod_j^D \frac{1}{2} \pi w^2 Z \end{aligned}$$

where

$$\begin{aligned} Z =& \frac{2}{\sqrt{\pi}} w \exp\left[-1/w^2 \right] \mathrm{erf}\left(1/w \right) + \mathrm{erf}^2\left(1/w \right) - \sqrt{\frac{2}{\pi}} w \mathrm{erf}\left(\sqrt{2}/w \right) \\ &+ \mathrm{erf}\left(\frac{1}{2w} \right) - \frac{2w}{\sqrt{\pi}} \left[1 - \exp\left[-\frac{1}{4w^2} \right] \right] \end{aligned}$$

which can be further simplified in the small w case to find

$$\sigma_V^2 \approx \frac{V^2}{N w^D \left[2\pi \right]^{D/2}} + \frac{V^2 \frac{\pi}{2}^D}{\left[\sqrt{\pi} - w \right]^{2D}} \left[2 - \frac{\sqrt{2}+2}{\sqrt{\pi}} w \right]^D - V^2$$

This expression has two parts: a decay with population size (with N, first term) and a stable value for arbitrarily large population size (the second two terms).
