# Supplementary material for: Redundant representations are required to disambiguate simultaneously presented complex stimuli
Source: PLoS Comput Biol. 2023 Aug 9;19(8):e1011327. doi: 10.1371/journal.pcbi.1011327 (PMC10442167; doi:10.1371/journal.pcbi.1011327)
Supplement: Attachment — Submitted filename: response.pdf [file pcbi.1011327.s002.pdf]

1 Description of main changes

We are grateful to both reviewers for their detailed and thoughtful engagement with our original manuscript. In response to their criticisms, comments, and clarification questions we have extensively rewritten sections of the introduction, results, and discussion. Among other things, these edits have been focused on improving clarity on several key points, and to contextualize our results and predictions more deeply in the wider neuroscience literature. As part of this, we have also added several new sections to these areas of the paper. As a result of these edits, we believe that the manuscript has been significantly improved.

Below, we have provided a detailed response to each reviewer comment as well as a reference to the line in the paper where the comment is addressed. We have reproduced all text from the reviews in blue; the text from our revised manuscript is given in green. The line numbers refer to lines in the tracked changes version of the manuscript. For ease of reference, the citations in quoted parts of our revised manuscript are reproduced at the bottom of the document here.

2 Detailed response to reviewers

2.1 Reviewer 1

> In this study, Johnston and Freedman proposed a theoretical solution for how distributed representations are integrated (the assignment problem): that one or more common stimulus features are represented in pairs of relevant brain regions. They further implemented the solution in a biological plausible receptive field code, and showed the tradeoff between assignment errors and redundancy. Lastly, they trained a feedforward neural network to show that the optimal solution can be learned when an integration layer was included.
>
> Overall, I think this is a technically sound study that addresses an important question in the field, and has potential implications for experimental work in different domains. Meanwhile, I have a few clarification questions:

We thank the reviewer for their thoughtful review.

2.1.1

> 1. This work focuses on theoretical solutions of the assignment problem, but I think it would be greatly helpful if the authors could provide more concrete examples, or more concrete predictions, of the solution. For instance, it is reasonable to expect such integrated representations to be found in multi-sensory associative regions, in the example of visual-auditory integration; but how exactly would common features develop and integrate in the case of multi-region representations within a single sensory system? From how I read it, the authors were referring to regions in different sensory pathways (e.g., the dorsal and ventral visual pathways, as in ref. 30); however, the authors also talked about regions within a sensory pathway (e.g., along the ventral visual stream, as in ref. 49). So, making it more explicit, and possibly discussing where in the brain to expect such integrated representations, could help.

We have included additional detail about and motivation for our predictions in an expanded section of the discussion:

> **(line 454)** Second, our work shows that explicit bound representations of the integrated and common features may be a necessary step in solving the assignment problem. Following previous work[1–3] and our neural network results, we believe that these bound representations will be instantiated through a conjunctive population geometry for the bound features – similar to the multi-dimensional receptive fields used above (though the representation need not have exactly this form). Thus, for a multi-sensory classification task, we expect to find these bound representations in multi-sensory association areas (such as posterior parietal cortex[4, 5], ventrolateral prefrontal cortex[6], and the superior temporal sulcus[7]; also see [8]). For complex visual decision-making tasks where integration across the dorsal and ventral visual streams is required, we expect to find bound representations in brain regions receiving input from both streams (such as prefrontal or posterior parietal cortex[9, 10]) as well as regions within each stream that receive information from the other stream. For instance, recent work has shown combined representations of visual form and motion in the inferotemporal cortex[11], though their geometry was not characterized (i.e., it is unknown whether the representations are bound). Similar integrated representation of visual form and motion have also been found in the superior temporal polysensory area[12] (and in the middle[13] and body-selective patches within[14] the superior temporal sulcus).
>
> Third, our framework also makes predictions that can be tested with population recordings from uni-sensory brain regions, during the performance of a multi-sensory task (or, at least, a task that is believed to involve assignment). Our work predicts that behavioral assignment errors – i.e., a swap error[2, 15, 16], described above – will be correlated with errors in the representation of the common features specifically in the direction of other stimuli. For instance, in a visual-auditory integration task with multiple stimuli, we expect that trials in which a position decoder for one of the stimuli makes errors toward another stimulus will also be associated with a greater probability of behavioral assignment errors. We do not expect errors in the representation of non-common features to have this same correlation with assignment errors – instead, we expect that they will be associated with local variance in the animal's report and would be detectable in continuous report tasks, such as [17].

We have also included additional discussion of the dorsal-ventral case of assignment (within the quote above), beginning on line 465.

2.1.2

> 2. In Section 2.4, the authors discussed two errors that contribute to combined MSE: local and threshold errors. The tradeoff between the two errors is critical in determining the optimization of MSE. However, in the following Section 2.5, all analyses were restricted to RF codes with negligible threshold error rates, making the assignment problem a lot simplified. I did not find the rationale for doing this, and I would like to see more clarification and discussion on this issue.

We thank the reviewer for their attention to this point. We now discuss how threshold errors interact with the assignment process in the paper,

> **(line 286)** Next, we link these RF codes to our theory of assignment errors. The local variance of the RF code is equivalent to the local variance in our framework. However, the threshold errors have no clear analogue in our current theory. Since the inferred stimulus produced from a threshold error is uniformly distributed across the whole stimulus space, they are extremely disruptive to assignment: The optimal strategy is not to integrate the threshold error representation during the assignment process at all. This unbalanced version of the assignment problem has been studied in combinatorics[18], but is beyond the scope of the current work. However, threshold errors are typically unlikely once the optimal RF width has been chosen. In particular, we find that codes with non-negligible threshold error rates also tend to have high total error. To proceed, we exclude codes with high total error, both due to the issues described for threshold errors and because our analytic calculation for the assignment error rate assumes local variance that is small relative to the size of the stimulus space.

We had initially excluded codes with non-negligible threshold error rates due to this complication. When we returned to include them, however, we found that the only codes that have high threshold error rates also have high local variance (MSE) and therefore high assignment error rates. All of the same codes are excluded when we exclude codes with high total error. We believe that this is necessary since our calculation of the assignment error rate requires local variance (MSE) that is small relative to the size of the stimulus space. Thus, we have excluded codes in section 2.5 with total error greater than half the size of the stimulus space, and noted this condition in the text,

> **(line 332)** Codes with total error that exceeds half the size of the stimulus space are excluded from this analysis for the reasons discussed above.

2.1.3

> 3. Variants of the assignment problem have been studied in previous literature, such as the feature binding problem (e.g., Schneegans & Bays, 2017), and the causal inference problem (e.g., Kording et al., 2007). Although these alternative models were briefly introduced in Introduction, a more thorough discussion on the similarities and differences between the current and previous models would still be necessary. For example, the paper discussed successful integration and incorrect integration (i.e., swap error as in feature binding), but how would segregation (e.g., separate sources of visual and auditory information as in causal inference) occur and quantified in the current solution?

The reviewer is correct that we have focused on what we call "balanced" assignment in this manuscript, where there is a single, correct one-to-one mapping between sets of representations of equal size. However, we agree that the unabalanced case is also interesting. We have added an expanded discussion of this case to the Discussion, with some notes about how our framework can be generalized to that case,

> **(line 534)** We have also discussed the case of balanced assignment between two brain regions, where each region represents the same number of stimuli and there is a correct one-to-one mapping between those stimuli. However, this is a simplification: in many cases, there may be more representations in one region than the other (e.g., speakers who are not visible). Or, even when there are the same number of representations, a one-to-one mapping may not be correct (e.g., a speaker behind the subject and another person in view who is not speaking). This latter situation has been studied for a single auditory

and single visual stimulus[19–21]. This work found evidence that human observers infer a single cause for the two percepts only when they are nearby in space; otherwise, they infer independent causes – and further shows that this process is well-modeled by Bayesian inference, depending on the local variance of auditory and visual position estimates as well as the prior rate of common relative to distinct causes[21]. Future work can combine our approach to the assignment problem with this inference of the number of distinct causes in the environment. This could be achieved by considering more potential mappings between the sets of representations, along with a prior giving the strength of the expectation that each representation will be integrated. This generalization of our framework would lead to additional predictions for experimental data with unbalanced numbers of stimuli across distinct sensory modalities.

We have also expanded our discussion of the alternative models,

> **(line 437)** This work extends previous work on the assignment problem in several ways. First, previous studies have considered either a single stimulus in both modalities (one auditory and one visual stimulus in [21]) or a single stimulus that must be assigned to one stimulus from a larger array (using a cue to select a single stimulus from a remembered array in [1, 2]). Here, we have shown how the rate of assignment errors scales as more stimuli need to be integrated. Second, previous work used a single common feature for binding[1, 2, 21], while we have shown how assignment error rates scale for additional common features. Third, the formulation of the neural code that we use is similar to that used in [2]; however, we have derived novel closed form expressions for the total, local, and threshold error rates of these codes – using these expressions we investigate a wider variety of different neural architectures (e.g., splitting representations across different regions) than have been considered in previous work. Finally, we believe that our neural network approach to producing assigned representations is novel, as previous work only characterized the error rates that would be expected from an ideal observer[1, 2, 21]. Thus, we believe that this work contributes to our understanding of the assignment problem, building on top of this foundational work.

2.2 Reviewer 2

> This manuscript describes a computational solution to the problem of assigning multiple properties (represented by distinct regions/modalities) to multiple objects, a problem related to causal inference in multisensory integration but with a slightly different twist. The approach seems elegant and creative, the results are interesting and the paper very well written. I think it should be published and will be of interest to many.

We thank the reviewer for their kind comments about our manuscript.

2.2.1

> However, although logically and mathematically sound, as a theory of brain function I found it somewhat lacking. The premise as explained out of the gate seems a little farfetched and possibly circular. It assumes a kind of object-level organization that may not exist in most sensory cortices, at least not early/primary areas. Labeling the relevant visual area V1 in Fig. 1A (instead of, say, IT) for high-level representations like dog/cat is of course incorrect (not sure about A1), which doesn't make a good first

impression. That's a minor thing, but I think the issue is deeper one, and relates to two poorly defined concepts: 'object' and 'region' (see below). I also have a third general comment about the theory's (in my view insufficient) contact with neurophysiology and anatomy.

We have changed the example given in the introduction as follows:

> **(line 39)** Coherent behavior in complex natural environments requires extensive and reliable integration of different forms of information about the world. For instance, a pedestrian crossing a crosswalk during rush hour attends to the flow of traffic around the intersection – if they hear honking, it can be important to quickly localize the honking cars and decide whether they need to change their motor plan. While navigating cluttered multisensory environments like this one often appears effortless for humans and other animals, it requires two highly non-trivial computations: Object segmentation and representation assignment.

We have also ammended the labels to be ITC and A1. We agree that this is more accurate for representations of animals. We agree also that the concepts of object and region are difficult and lead to some confusion and apparent circularity. We have also clarified what we mean by brain region:

> **(line 160)** Finally, throughout this work we organize our discussion using the concept of distinct brain regions, by which we mean distinct populations of neurons that may share information with each other through anatomical connections. We primarily use this concept for simplicity of exposition. In general, all of the same principles apply when different features (or combinations of features) are represented in distinct subspaces of the same neural population activity. That is, the assignment problem will also need to be solved to integrate across these distinct subspaces, even though the subspaces do not correspond to distinct populations of neurons.

In addition, we now emphasize that these assigned representations are necessary for decision-making in the presence of multiple options:

> **(line 363)** In general, we believe that the assignment problem is solved to make decisions that require the linking of two or more stimulus features (e.g., asking what the value of feature X is for stimuli with a particular value of feature Y, as in the human working memory studies discussed above).

This contextualization of the assignment problem in a decision-making process is now mentioned in other parts of the paper as well.

We do believe that assigned representations of certain (particularly decision-relevant) features are necessary to avoid the ambiguity arising from multiple representations that we describe in the paper, but we agree that platonic object representations may not exist. We have attempted to clarify that throughout without compromising clarity of exposition.

2.2.2

> 1. First, the conception of the assignment problem assumes that the segmentation and binding problems (within a modality) have already been solved, and all that remains is to assign features to the appropriate objects. I wrote a long diatribe about this before

> realizing that the paper covers it concisely in the Discussion — shame on me for not reading all the way through first, but it means that many readers will have this impression too, and you should confront this limitation earlier, i.e. in the Introduction. I still think it might weaken the premise due to circularity, since one could argue segmentation and binding already require a solution to the assignment problem. But I suppose that's no reason to hold up publication; the community can be the judge.

We agree that this is important to clarify. We have added a note about representations of bound features to the Introduction,

> **(line 114)** In the feedforward network, we also show how the integrated stimuli can be reliably represented: through nonlinearly mixed representations of the integrated features, which follows from previous work on representations of multiple stimuli[1–3].

We believe that assignment, segmentation, and binding are all interleaved and now discuss this more in the manuscript,

> **(line 522)** The simultaneous representation of multiple stimuli in neural activity is not fully understood. In particular, while we have provided a solution to the assignment problem, which must be solved when integrating representations of multiple stimuli that are distributed across different brain regions, distinct neural population subspaces within a single brain region, or even time points. However, the assignment problem is only one of several difficulties that arise when multiple stimuli are represented simultaneously. One additional problem is the segmentation, or clustering, of sensory information into a discrete set of causes (i.e., stimuli or objects). While there is extensive work on this process in psychology[22–24] and in machine learning[25, 26], the neural mechanisms are not fully understood (but see [27, 28]). A second additional problem that we have already mentioned is the representation of bound stimulus features in a single population of neurons; in this work, we have assumed that this representational problem has already been solved, perhaps by the conjunctive feature representations posited by other work[1–3] (but see [29–32] for alternatives). In the real brain, each of these problems is likely solved at many different stages of sensory processing (or even simultaneously), where lower-level components are clustered into higher-level features (e.g., combining points into an edge), a correct binding of those features is inferred (e.g., that edge is combined with a representation of its motion), then that bound set of features is represented (and can become a new, higher-level feature itself – e.g., perhaps the edge is part of a running dog). While we have focused on one part of this process, future work can integrate these parts and more fully explore how they interact – both with each other, and with other concerns (such as how multiple representations in a single neural population interact with each other, see [33]). This unifying work will be necessary to develop a full understanding of the rich ways that simultaneously representing multiple stimuli constrains the neural code.

2.2.3

> 2. Second, the notion of a 'brain region' (especially critical for section 2.5), and its relationship to perception, is underdeveloped. The authors write: "The assignment problem must be solved when stimulus features are represented in distinct brain regions" — Why? Because perception requires convergence of information onto a single region

(or single neurons)? That's a big assumption. What region might that be? Isn't our modern understanding that perception and behavior emerge from the collective activity of many interconnected populations/regions? If so, it becomes harder to motivate the problem, I think.

And even if 'region' is the right level of anatomical organization to think about here, how do you define the term? Neural populations are separated spatially but connected synaptically—do two highly interconnected but spatially disparate populations count as one region? What about two very nearby but only weakly connected populations? Or is a region defined by a complete representation of some stimulus dimension, like how boundaries between visual and somatosensory areas are defined by where the retinotopic or somatotopic map mirror-flips and begins to repeat? This works for most early sensory cortices but probably not later ones (e.g. where 'dog' and 'cat' live).

We agree that brain regions are a slippery concept, and have included a working definition in the text (also quoted above),

> **(line 160)** Finally, throughout this work we organize our discussion using the concept of distinct brain regions, by which we main distinct populations of neurons that may share information with each other through anatomical connections. We primarily use this concept for simplicity of exposition. In general, all of the same principles apply when different features (or combinations of features) are represented in distinct subspaces of the same neural population activity. That is, the assignment problem will also need to be solved to integrate across these distinct subspaces, even though the subspaces do not correspond to distinct populations of neurons.

We have also discussed how assignment can unfold over time instead of across distinct brain regions,

> **(line 486)** In general, we expect that these intermediate representations will emerge prior to a pure representation of the decision variable – both in brain regions that are earlier in an established processing hierarchy as well as temporally earlier within brain regions that ultimately express representations of the decision variable. While the results from our neural network study underline the importance of this bound representation for computing the decision variable (Figure 5), a single neural population could simultaneously represent these distinct forms of information in separate subspaces of activity. These bound representations have also been identified as important for representation recoding[34]. Future work could identify whether these recoding and assignment operations can be performed simultaneously in the same integrated representation.

In general, we agree that the collective responses of populations of neurons (which need not be in a particular anatomical region) are the important unit of computatation – and we believe this is consistent with transitioning from an non-assigned representations (which have a certain geometry) and assigned representations (which have a different geometry). We discuss these geometries more in the discussion as well,

> **(line 472)** Following previous work[1–3] and our neural network results, we believe that these bound representations will be instantiated through a conjunctive population geometry for the bound features – similar to the multi-dimensional receptive fields used above (though the representation need not have exactly this form).

This same section is also quoted above.

The point is not that this paper needs to solve these quasi-philosophical issues (although there are some philosophers out there who might be worth consulting). But it could be made clearer what is assumed about the 'read-out' of neural activity that necessitates the solution you propose. In other words, after the assignment problem is solved, what sort of neural representation are you left with, and what is the next step in the causal chain to behavior?

In the new sections quoted above, we have emphasized the geometry of the assigned neural representation as well as the fact that assigned representation can then be transformed into the representation of a decision variable, which can then be used to guide a decision.

2.2.4

3. Lastly, the theory could be better linked to the neurobiology, assuming that is a goal the authors seek. Aspects of this issue have been touched on above: defining regions better and being more up-front about the requirement for object-level representations (implicating later stages of processing). But when it comes to specific predictions for experiments, the relevant paragraph in Discussion (387-396), although a good start, comes up short.

For instance, "Brain regions that commonly represent multiple stimuli will all have some redundancy with other brain regions" — that's a bit vague and probably trivially true (or already known to be true), therefore not very useful as a prediction.

And, "Our framework can be used to predict assignment error rates based on the level of redundancy between two brain regions" — how exactly would that play out? Can you be more specific?

We agree that the "level of redundancy" statement is a little too vague – and relies in part on an approach that we removed from the manuscript prior to submission for coherence, so we have removed it.

We have worked to make our other predictions more precise, as well as expanded on some more specific analysis approaches. Such as,

(line 459) In some cases, the redundant information between two regions may be clear due to experimental design. However, in cases where redundant features are not readily identifiable, population analyses can be used to discover redundant information directly by building models that explain the activity in one region in terms of the activity in the other. Then the activity explained by this approach can be modeled in terms of known experimental variables. For instance, in this way, common features across the parallel ventral and dorsal visual streams in the primate visual system can be discovered (though some features, such as spatial position, have already been shown to be redundant across the two streams[35]).

and

(496) Third, our framework also makes predictions that can be tested with population recordings from uni-sensory brain regions, during the performance of a multi-sensory task (or at least a task that is believed to involve assignment). Our work predicts that behavioral assignment errors – i.e., a swap error[2, 15, 16], described above – will

be correlated with errors in the representation of the common features specifically in the direction of other stimuli. For instance, in a visual-auditory integration task with multiple stimuli, we expect that trials in which a position decoder for one of the stimuli makes errors toward another stimulus will also be associated with a greater probability of behavioral assignment errors. We do not expect errors in the representation of non-common features to have this same correlation with assignment errors – instead, we expect that they will be associated with local variance in the animal's report and would be detectable in continuous report tasks, such as [17].

We have also been more specific about regions expected to have the assigned representations we describe, in both the multisensory and visual contexts,

(line 475) Thus, for a multi-sensory classification task, we expect to find these bound representations in multi-sensory association areas (such as posterior parietal cortex[4, 5], ventrolateral prefrontal cortex[6], and the superior temporal sulcus[7]; also see [8]). For complex visual decision-making tasks where integration across the dorsal and ventral visual streams is required, we expect to find bound representations in brain regions receiving input from both streams (such as prefrontal and posterior parietal cortex[9, 10]) as well as regions within each stream that receive information from the other stream. For instance, recent work has shown combined representations of visual form and motion in the inferotemporal cortex[11], though their geometry was not characterized (i.e., it is unknown whether the representations are bound). Similar integrated representation of visual form and motion have also been found in the superior temporal polysensory area[12] (and in the middle[13] and body-selective patches within[14] the superior temporal sulcus).

What about RF codes? Can you say anything about (for example) how the width of RFs in real data compare to what is 'optimal' or predicted by the model? This would probably require defining a behavioral task and feature space in more concrete terms, but some intuitive/qualitative attempt at this could be valuable and increase the interest level in the field.

While we do not feel comfortable making a specific prediction related to RF width (due to its dependence on many factors outside of assignment), we have emphasized our prediction about the conjunctive nature of the potentially RF-like codes underlying assigned representations,

(line 472) Following previous work[1–3] and our neural network results, we believe that these bound representations will be instantiated through a conjunctive population geometry for the bound features – similar to the multi-dimensional receptive fields used above (though the representation need not have exactly this form).

I gather that the manuscript overall is written intentionally to be as general as possible, but in the name of generality it may have missed an opportunity for more concrete biologically informed linkages/predictions that will excite experimentalists and theorists alike.

We hope that our changes to this section of the manuscript (many of which are reproduced above) has better navigated this tradeoff between generality and specificity.

2.2.5 Specific/Minor Comments

line 12 — Honestly, I'd go with 'meowing' :). I mean you can barely hear a purr, certainly not from very far away, and it's usually associated with being stroked or contented in some other manner. [Oh, I see you already made this choice in Fig 1 and elsewhere—just have to change it in the abstract and line 125 for consistency.]

We have made this change.

line 90, and throughout — Why use MSE instead of variance (more common in the field?), given that the estimates are assumed to be unbiased?

We have changed over to local variance instead of local MSE.

Figure 1F — can't really see the dashed lines.

We have replotted this panel (and other panels with dashed lines) to make them more visible by the inclusion of a black outline around the dashes.

Figure 2D — why does the graph get all janky towards the bottom-right?

This was due to not having enough samples for that error rate. We have re-generated the panel with more samples.

line 244 — what does Fisher Info have to do with metabolic cost?

We have clarified this line,

(line 260) To see this, we begin by deriving the average Fisher information of a random RF code with a particular number of neurons and a particular average level of activity across the whole population (i.e., a budget of metabolic energy to be used for spiking)...

line 592 — "there is evidence" - can you cite some here?

We have added citations to this statement – specifically to some of the work on the two primate ventral and dorsal visual streams.

Several figures are described as comparing theory (analytical calculations?) with simulations, but I don't recall seeing where it is explained how the simulated data are generated. Do they come from a generative process described by the equations, and if so, isn't it guaranteed that they would match the 'theory' in the limit of large Nsims?

We have added sections to the methods explaining how the simulations assignment error and RF code error simulations are done. If our analytical calculations are correct and our simplifications are appropriate, then there should be perfect agreement between the lines (and indeed this is more or less what we find). Since we do make several simplifications in the course of the analytical calculations, though, we thought it was helpful to include the simulations to reassure the reader (and ourselves).

References

1. Matthey, L., Bays, P. M. & Dayan, P. A probabilistic palimpsest model of visual short-term memory. *PLoS computational biology* **11,** e1004003 (2015).
2. Schneegans, S. & Bays, P. M. Neural architecture for feature binding in visual working memory. *Journal of Neuroscience* **37,** 3913–3925 (2017).

3. Johnston, W. J., Fine, J. M., Yoo, S. B. M., Ebitz, R. B. & Hayden, B. Y. Subspace orthogonalization as a mechanism for binding values to space. *arXiv preprint arXiv:2205.06769* (2022).
4. Mullette-Gillman, O. A., Cohen, Y. E. & Groh, J. M. Eye-centered, head-centered, and complex coding of visual and auditory targets in the intraparietal sulcus. *Journal of neurophysiology* **94,** 2331–2352 (2005).
5. Avillac, M., Hamed, S. B. & Duhamel, J.-R. Multisensory integration in the ventral intraparietal area of the macaque monkey. *Journal of Neuroscience* **27,** 1922–1932 (2007).
6. Sugihara, T., Diltz, M. D., Averbeck, B. B. & Romanski, L. M. Integration of auditory and visual communication information in the primate ventrolateral prefrontal cortex. *Journal of Neuroscience* **26,** 11138–11147 (2006).
7. Barraclough, N. E., Xiao, D., Baker, C. I., Oram, M. W. & Perrett, D. I. Integration of visual and auditory information by superior temporal sulcus neurons responsive to the sight of actions. *Journal of cognitive neuroscience* **17,** 377–391 (2005).
8. Stein, B. E. & Stanford, T. R. Multisensory integration: current issues from the perspective of the single neuron. *Nature reviews neuroscience* **9,** 255–266 (2008).
9. Cavada, C. & Goldman-Rakic, P. S. Posterior parietal cortex in rhesus monkey: II. Evidence for segregated corticocortical networks linking sensory and limbic areas with the frontal lobe. *Journal of Comparative Neurology* **287,** 422–445 (1989).
10. Webster, M. J., Bachevalier, J. & Ungerleider, L. G. Connections of inferior temporal areas TEO and TE with parietal and frontal cortex in macaque monkeys. *Cerebral cortex* **4,** 470–483 (1994).
11. Burk, D. C. & Sheinberg, D. L. Neurons in inferior temporal cortex are sensitive to motion trajectory during degraded object recognition. *Cerebral Cortex Communications* **3,** tgac034 (2022).
12. Oram, M. & Perrett, D. Responses of anterior superior temporal polysensory (STPa) neurons to "biological motion" stimuli. *Journal of cognitive neuroscience* **6,** 99–116 (1994).
13. Tanaka, Y. Z., Koyama, T. & Mikami, A. Visual responses in the temporal cortex to moving objects with invariant contours. *Experimental brain research* **146,** 248–256 (2002).
14. Jastorff, J., Popivanov, I. D., Vogels, R., Vanduffel, W. & Orban, G. A. Integration of shape and motion cues in biological motion processing in the monkey STS. *Neuroimage* **60,** 911–921 (2012).
15. Bays, P. M., Catalao, R. F. & Husain, M. The precision of visual working memory is set by allocation of a shared resource. *Journal of vision* **9,** 7–7 (2009).
16. McMaster, J. M., Tomić, I., Schneegans, S. & Bays, P. M. Swap errors in visual working memory are fully explained by cue-feature variability. *Cognitive psychology* **137,** 101493 (2022).
17. Panichello, M. F. & Buschman, T. J. Shared mechanisms underlie the control of working memory and attention. *Nature* **592,** 601–605 (2021).
18. Burkard, R., Dell'Amico, M. & Martello, S. Assignment Problems. *SIAM* (2009).
19. Hairston, W. D. *et al.* Visual localization ability influences cross-modal bias. *Journal of cognitive neuroscience* **15,** 20–29 (2003).
20. Wallace, M. T. *et al.* Unifying multisensory signals across time and space. *Experimental brain research* **158,** 252–258 (2004).
21. Körding, K. P. *et al.* Causal inference in multisensory perception. *PLoS one* **2,** e943 (2007).
22. Köhler, W. Gestalt psychology. *Psychologische Forschung* **31,** XVIII–XXX (1967).
23. Spelke, E. S. Principles of object perception. *Cognitive science* **14,** 29–56 (1990).

24. Wagemans, J. *et al.* A century of Gestalt psychology in visual perception: I. Perceptual grouping and figure–ground organization. *Psychological bulletin* **138,** 1172 (2012).
25. Cheng, B. *et al. Panoptic-deeplab: A simple, strong, and fast baseline for bottom-up panoptic segmentation* in *Proceedings of the IEEE/CVF conference on computer vision and pattern recognition* (2020), 12475–12485.
26. Chen, H. *et al.* Unsupervised Segmentation in Real-World Images via Spelke Object Inference. *arXiv preprint arXiv:2205.08515* (2022).
27. Zhou, H., Friedman, H. S. & Von Der Heydt, R. Coding of border ownership in monkey visual cortex. *Journal of Neuroscience* **20,** 6594–6611 (2000).
28. Roelfsema, P. R. *et al.* Cortical algorithms for perceptual grouping. *Annual review of neuroscience* **29,** 203–227 (2006).
29. Gray, C. M., König, P., Engel, A. K. & Singer, W. Oscillatory responses in cat visual cortex exhibit inter-columnar synchronization which reflects global stimulus properties. *Nature* **338,** 334–337 (1989).
30. Martin, A. B. & von der Heydt, R. Spike Synchrony Reveals Emergence of Proto-Objects in Visual Cortex. *Journal of Neuroscience* **35,** 6860–6870. ISSN: 0270-6474. `http://www.jneurosci.org/content/35/17/6860.short` (2015).
31. Caruso, V. C. *et al.* Single neurons may encode simultaneous stimuli by switching between activity patterns. *Nature communications* **9,** 2715 (2018).
32. Jun, N. Y. *et al.* Coordinated multiplexing of information about separate objects in visual cortex. *Elife* **11,** e76452 (2022).
33. Orhan, A. E. & Ma, W. J. Neural population coding of multiple stimuli. *Journal of Neuroscience* **35,** 3825–3841 (2015).
34. Deneve, S., Latham, P. E. & Pouget, A. Efficient computation and cue integration with noisy population codes. *Nature Neuroscience* **4,** 826–831. ISSN: 1097-6256. `http://www.ncbi.nlm.nih.gov/pubmed/11477429` (2001).
35. Hong, H., Yamins, D. L. K., Majaj, N. J. & DiCarlo, J. J. Explicit information for category-orthogonal object properties increases along the ventral stream. *Nature Neuroscience* **19,** 613–622. ISSN: 1097-6256. `http://www.nature.com/doifinder/10.1038/nn.4247%7B%5C%%7D5Cnhttp://www.ncbi.nlm.nih.gov/pubmed/26900926` (2016).
